# Embodying functionally relevant action sounds in patients with spinal cord injury

**DOI:** 10.1038/s41598-018-34133-z

**Published:** 2018-10-23

**Authors:** Mariella Pazzaglia, Giulia Galli, James W. Lewis, Giorgio Scivoletto, Anna Maria Giannini, Marco Molinari

**Affiliations:** 1 Dipartimento di Psicologia, University of Rome ‘La Sapienza’, Via dei Marsi 78, 00185 Rome, Italy; 2 IRCCS Fondazione Santa Lucia, Via Ardeatina 306, 00179 Rome, Italy; 3 Department of Neurobiology and Anatomy, West Virginia University, Morgantown, West Virginia USA; 4 Center for Advanced Imaging, West Virginia University, Morgantown, West Virginia USA

## Abstract

Growing evidence indicates that perceptual-motor codes may be associated with and influenced by actual bodily states. Following a spinal cord injury (SCI), for example, individuals exhibit reduced visual sensitivity to biological motion. However, a dearth of direct evidence exists about whether profound alterations in sensorimotor traffic between the body and brain influence audio-motor representations. We tested 20 wheelchair-bound individuals with lower skeletal-level SCI who were unable to feel and move their lower limbs, but have retained upper limb function. In a two-choice, matching-to-sample auditory discrimination task, the participants were asked to determine which of two action sounds matched a sample action sound presented previously. We tested aural discrimination ability using sounds that arose from wheelchair, upper limb, lower limb, and animal actions. Our results indicate that an inability to move the lower limbs did not lead to impairment in the discrimination of lower limb-related action sounds in SCI patients. Importantly, patients with SCI discriminated wheelchair sounds more quickly than individuals with comparable auditory experience (i.e. physical therapists) and inexperienced, able-bodied subjects. Audio-motor associations appear to be modified and enhanced to incorporate external salient tools that now represent extensions of their body schemas.

## Introduction

The notion of embodied cognition postulates that knowledge is grounded on actual bodily states and that higher-order processes, such as mind- and intention- reading or action- and perception- understanding, can be mapped onto modal sensorimotor cortices^1^. The bodily instantiation of cognitive operations, or “embodiment”, and perceptual-motor state, or “simulation”, are thought to enable the inter-individual sharing of experiences^2^. Based on the results of single-cell recordings in monkeys^3,4^, many neuroimaging and neurophysiological studies have proposed that the adult human brain is equipped with neural systems and mechanisms that affect the perception and execution of actions in a common format^5–8^. Direct action perception strengthens motor representation^9^, and short-term motor experiences with a particular action may influence its visual recognition^10^ and facilitate action prediction^11^.

Both visual and auditory channels participate in perception-action coupling^12^. The mechanisms and neural structures involved in the motor coding of action-related sounds have been explored in able individuals using correlative and causative approaches^13–18^. These studies indicate that the perception of sounds from body part specific actions (e.g. ripping a sheet of paper) activates the left fronto-parietal network^19^ in a somatotopic arrangement^16,20^. Moreover, greater involvement of the left *vs*. right inferior parietal lobe has been reported when an observer’s attention is explicitly directed toward action sounds^21^.

The inability to perform or perceive a given motor action may impact on the structural integrity of that action representation^22^. In patients with apraxia, impairment in specific actions execution (e.g. inability to clap the hands) greatly reduces the individual’s capacity to acoustically recognize the corresponding motor event. However, the inadequate discrimination critically depends on a properly functioning left fronto-parietal network^23^. Individuals with congenital blindness and deafness who have total perceptual loss rely on less implicit motor representations when perceiving human actions^24–26^. Blind people, however, may still rely on the coding of aural tool action via simulation, which could reflect the activity of an inherent motor system^24,26^. This pattern of results suggests that the failure to “capture” the sensorial or motor information hinders the processes of auditory mapping of actions. Individuals with spinal cord injury (SCI) who are unable to move their lower limbs, have a reduced ability to discriminate between different observed movements, suggesting that action mapping may be fully determined by immediate motor signals^27,28^. However, recent data suggested the original representation of the limb deafferented persists in the sensorimotor cortex, even decades after deafferentation^29–31^. Thus, the origins of reduced perceptual sensitivity for biological motion are very unclear.

This functional imbalance of perceptual motor states may be partially restored with active tool use^32^. A body-held tool, for example, may become essential to the user if it facilitates mobility or other essential functions^33,34^. When this is the case, the tool may be processed as a part of one’s own body^35–38^ and guide visual-motor^39^ and audio-motor^33,40^ interactions. Indeed, people with SCI who are paralyzed and wheelchair-bound could treat their relevant artificial tools (wheelchair) as an extension/substitution of the functionality of the affected body part^41^.

In principle, individuals with SCI may be ideal for testing two fundamental, largely unaddressed simulation and embodiment issues: (*i*) how motor afference/efference influences the functional integrity of audio-motor mapping; and (*ii*) how relevant extracorporeal tools (e.g. wheelchairs) affect action representations.

We hypothesize that the perceptual and motor experiences induced by the sounds of a wheelchair and lower limb activity should differ substantially between subjects with different levels of exposure to wheelchair- and limb- action. To test this hypothesis, we examined audio-motor mapping in three groups of participant. Wheelchair-bound patients with SCI have extensive motor and auditory experience of wheelchair sounds^42^, but do not have motor use of their legs. Physical therapists with normal limb function have extensive perceptual experience of wheelchairs, but are not personally motor dependent on them. The third group consisted of able-bodied controls that had no previous experience of wheelchairs. We devised a novel psychophysical task that evaluated the auditory discrimination ability of sounds originating from actions produced by wheelchair use, the upper and lower limb, and animals. Listening to sounds of various actions performed by a tool or lower limbs allowed us to dissociate the perceptual and motor contributions of biological or artificial mobility entities^18^. Furthermore, the given task allowed us to investigate the inverse relationship between movements that the patients had previously possessed, lost, and then regained with wheelchair use, providing a way to move and to act in the world again.

## Methods

### Participants

At the Santa Lucia Hospital in Rome, Italy, we recruited 20 subjects with established lumbar or thoracic SCI (17 men; mean age, 40.8 years; range, 19–56 years), 20 able-bodied participants who had worked exclusively with SCI patients as physical therapists (18 men; mean age, 39.9 years; range, 27–54 years), and 20 able-bodied subjects who were not physical therapists (12 men; mean age, 40.8 years; range, 20–66 years). The three groups did not differ in age and level of education (p > 0.95). The physical therapists were employed full-time and had an average of five years experience in SCI patient rehabilitation (range, 1–25 years). All of the subjects were right-handed, as determined by the 10-item version of the Edinburgh Handedness Inventory^43^. No participants presented auditory discrimination deficits or signs of psychiatric disorders, and none had a history of substance abuse. Written, informed consent was obtained from each participant for all procedures. The experimental protocol was approved by the ethics committee of the Fondazione Santa Lucia and was performed in accordance with the relevant guidelines and regulations of the 1964 Declaration of Helsinki.

### Assessment of individuals with SCI

All of the patients had a traumatic lesion at the thoracic or lumbar level of the spinal cord that caused paralysis of the lower limbs while sparing upper limb function. Lesions were located between T3 and L1, and the patients ranged from 6.3 to 219 months post-SCI (mean, 65 ± 75 months). Each patient was examined by a neurologist (G.S.) with specific, long-standing expertise in treating SCI patients. The neurological injury level was determined using the American Spinal Injury Association (ASIA) for the classification of SCIs^44^. Functional ability was quantified using the third version of the Spinal Cord Independence Measure (SCIM III)^45^. For the purposes of the experiment, the Self-care and the Management and Mobility subscales were considered. All patients were manual wheelchair users and recruited from physiotherapy programs of Spinal Cord Unit. None of the patients had experienced head or brain lesions, as documented by an MRI. The demographic and additional clinical data of the patients are presented in Table 1.

**Table 1:** Clinical and demographic data of the spinal cord injury patients.

Case	Age	Time since injury (days)	Gender	Lesion level	Etiology	AIS grade	SCIM		Motor level		Sensory level	
							Self Care	Mobility	Right	Left	Right	Left
P_1_	19	589	M	T10	Traumatic	A	20	19	T10	T10	T10	T10
P_2_	42	190	M	L1	Traumatic	A	20	18	L1	L1	L1	L1
P_3_	42	760	M	T10	Traumatic	A	20	19	T10	T10	T10	T10
P_4_	42	970	M	T9	Neoplastic	A	20	18	T9	T9	T6	T9
P_5_	35	4745	M	T8	Traumatic	A	20	19	T8	T8	T8	T8
P_6_	35	6570	M	T7	Traumatic	A	20	19	T7	T7	T7	T7
P_7_	49	320	M	L1	Traumatic	A	17	13	L1	L1	L3	L3
P_8_	42	390	M	T10	Traumatic	A	20	19	T11	T11	T11	T11
P_9_	38	240	F	T12	Traumatic	A	18	15	T12	T12	T12	T12
P_10_	44	365	M	T12	Traumatic	A	20	19	T12	T12	T12	T12
P_11_	39	970	M	T3	Traumatic	A	18	15	T6	T6	T6	T6
P_12_	36	1825	M	T12	Traumatic	A	20	19	T12	T12	T12	T12
P_13_	40	5840	M	T5	Traumatic	A	20	19	T5	T5	T5	T5
P_14_	42	3650	F	T5	Traumatic	A	20	19	T5	T5	T5	T5
P_15_	47	4330	M	T7	Traumatic	A	17	19	T8	T8	T6	T8
P_16_	54	1580	M	T10	Traumatic	A	20	18	T12	T12	T10	T10
P_17_	36	1270	M	T10	Traumatic	A	20	19	T10	T10	T10	T10
P_18_	56	1440	M	T7	Traumatic	A	20	19	T9	T9	T7	T7
P_19_	22	930	M	T12	Traumatic	A	20	18	T12	T12	T12	T12
P_20_	56	3650	F	T5	Traumatic	A	20	19	T5	T5	T5	T5

The clinical neurological level of the lesion (T, thoracic; L, lumbar) was reported for the subjects with spinal cord injury (SCI). The neurological and functional levels of the injury were determined using the American Impairment Scale (AIS) and the third version of the Spinal Cord Independence Measure (SCIM III). The motor/sensory level indicates the most caudal segment of the spinal cord with normal motor/sensory function.

### Sound-into-action translation test

Because the auditory system is an intact sensory channel to individuals with paraplegia, we used a sound-into-action translation task to explore the effects of a massive loss of motor function in the lower extremities on the ability to distinguish between different action-sounds. In a two-choice, matching-to-sample auditory action discrimination task, the participants were asked to determine which of two probe sounds matched the previously heard single sample sound. The sounds used included upper (U_RAS_) and lower (L_RAS_) limb-related action sounds, wheelchair-related action sounds (W_RAS_), and no human (animal) action-related sounds (NH_RAS_).

### Stimuli and task

The auditory stimuli (44.1 kHz, 16 bit, and monophonic) included 120 real-world sounds compiled by a sound engineer using professional collections (Sound Cinecittà, Rome, Italy, and Sound Ideas, Richmond Hill, Ontario, Canada). Many of these sounds were identical to those used in our previous studies^23,26^. The sounds were trimmed to an average duration of 4 sec (range, 3–6.5 sec) and presented to the participants at a comfortable decibel level through Sennheiser PC165 earphones, using the Presentation software (version 12.2, Neurobehavioral Systems, Inc.) on a Windows operating system.

Each sound belonged to one of the following four categories:

(1) Upper limb actions: a group of 10 sets of three different sounds. In this category, the sample, the matching and the non-matching stimuli were sounds of meaningful actions executed by the hands (e.g. knocking on a door).

(2) Lower limb actions: a group of 10 sets of three different sounds. In this category, the stimuli were three sounds of meaningful actions executed by the feet (e.g. descending footsteps on stairs).

(3) Wheelchair actions: a group of 10 sets of three different sounds. In this category, the stimuli were sounds of meaningful actions executed by manual or electronic wheelchair actions (e.g. WHC braking) and manual/electrical vehicle motion (e.g. bicycle braking).

(4) Non-human animal actions: a group of 10 sets of three sounds related to animal physical actions, excluding vocalizations (e.g. a bird flying).

A list of the auditory stimuli and information on the preliminary psychophysical studies are provided in the Supplementary Material.

### Procedures

**Figure 1:**
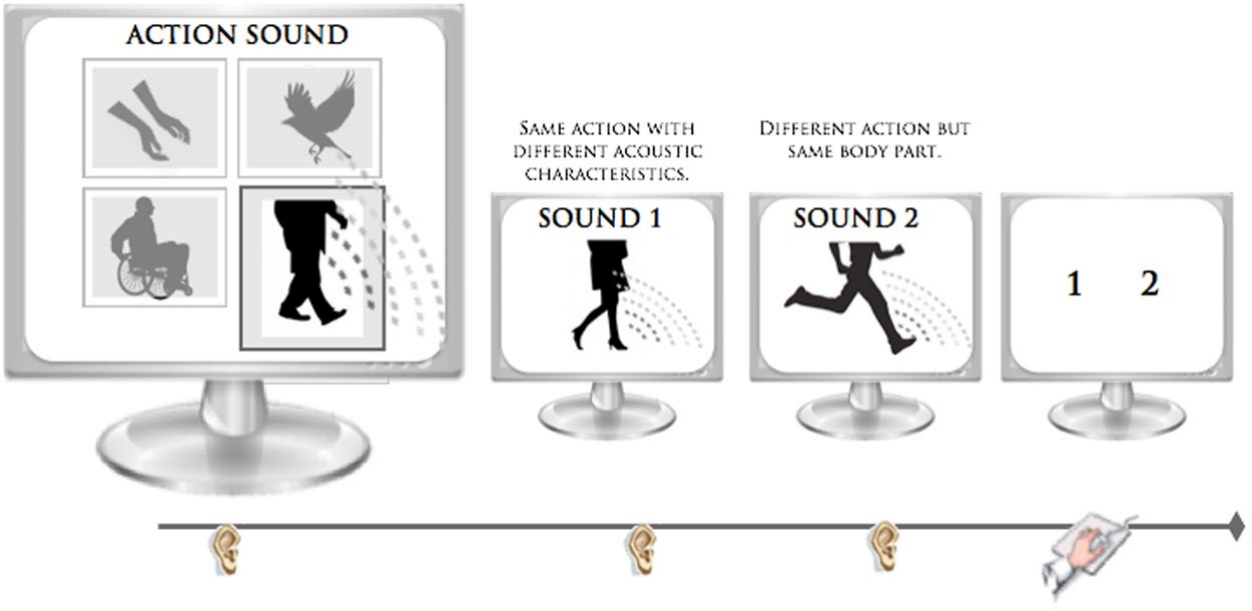
Action sound discrimination task. In each trial, following the presentation of a sample sound, two subsequent probe sounds were presented. Only one of the two probe sounds was specifically related to the sample sound. In the set of lower limb actions (e.g. “male footsteps on a glass surface” [the sample sound]), one probe sound represented the same action as the sample sound but was produced using a different source (e.g. “female footsteps on a wood surface”), whereas the other probe sound represented a totally different action produced using the same body part (e.g. “running”). No image associated with an aural action was provided.

Each participant was tested in a single experimental session that lasted approximately 20 minutes. During this period, the subjects wore earphones and sat approximately 50 cm from a 17-inch computer monitor. Each trial was initiated by the presentation of a sample action sound that was selected randomly from one of the four categories (i.e. U_RAS_, L_RAS_, W_RAS_ or NH_RAS_). At the end of the sample sound presentation, two subsequent matching and non-matching action-sound stimuli were presented in quick succession, separated by an approximate interval of 100 msec. The matching action-sound represented the same motor act as the sample, but with different acoustic features. The non-matching action-sound was acoustically similar to the sample, but linked to a different action within the same category. The sequence of matching and non-matching sound stimuli was counter balanced. For example in the U_RAS_ category, a brief sample sound of an individual clapping three times was presented, after which the subjects listened to two additional sounds, one of which represented the same action as the sample sound, but was produced using a different source (e.g. group applause) and the other sound represented a completely different action produced by using the same body part, which was acoustically similar (e.g. knocking on a door three times). A schematic representation of the human lower limb auditory stimuli and procedure is shown in Fig. 1.

The subjects were asked to choose between the two auditory stimuli to identify the sound that evoked the same action heard in the sample sound. To better discriminate among the three different sounds, the words “action sound” (for the sample sound) and the numerals 1 (for the first probe sound) and 2 (for the second probe sound) appeared on the black screen while each respective sound was played. No image associated with the aural action was provided. After all three sounds were presented, the final screen prompted the subject to choose a response by pressing a button. The participants were instructed to answer as accurately and quickly as possible, and their accuracy and response times after the prompt (i.e. latency) were recorded and analyzed. Before beginning the test, the participants were given four practice trials, after which performance feedback was provided. The practice auditory stimuli differed from those used in the experimental phase, after which no feedback was provided.

To evaluate the subjective rating of each sound category, a post-test session was conducted, in which the same participants were instructed to rank each sound in terms of familiarity and perceived motor intensity on a vertical 10-cm visual analog scale (VAS). The first question was intended to assess their experience with each sound category (“How familiar is this sound to you?”), while the second investigated a subject’s experience with the amount of movement sensations triggered by each sound (“To what degree do you feel your own movement is based on the action you have just heard?”). With regard to the first question, the lower and upper extremes of the VAS were “no familiarity” and “high familiarity,” respectively, whereas for the second question, these extremes indicated “no perceived movement” and “maximum perceived movement,” respectively. The participants were explicitly asked to rate the sounds, which were presented randomly, in a counterbalanced order. Finally, we collected structured reports on the implicit and explicit introspective experiences of regular wheelchair use in patients with SCI, using an adapted and slightly modified version of an ad-hoc devised questionnaire^41^.

### Data analyses

The accuracy (raw data) and mean latency were calculated for each participant in each experimental condition (10 trials per category). Trials in which the reaction times (RTs) were two or more standard deviations above the mean for each subject were eliminated prior to the analysis (2% of the trials)^46^. Half of the eliminated trials were associated with U_RAS_ actions. Only the RTs for the correct response were considered. The individual accuracy, mean latency values and subjective ratings were entered into separate mixed-model analyses of variance (ANOVAs), with group (healthy subjects, patients with SCI, and physical therapists) as the between-subjects factor and sound category (U_RAS_, L_RAS_, W_RAS_, and NH_RAS_) as the within-subjects factor. All pair-wise comparisons were performed using the Duncan *post hoc* test. The partial eta-squared (ηp2) measure of variance was selected as the index of effect size^47^. A significance threshold of p < 0.05 was set for all of the statistical analyses. The data are reported as mean ± standard error of the mean (SEM).

## Results

### Sound-into-action translation test

Sound discrimination performance in patients with SCI was >83% for all conditions. No significant effects of group (*F*_2,57_ = 1.17, p = 0.31), of sound category (*F*_3,171_ = 1.77, p < 0.15), and no group × sound category interactions (*F*_6,171_ = 1.06, p = 0.4) were observed, indicating that the three groups had comparable performance in the four different sound categories.

**Figure 2:**
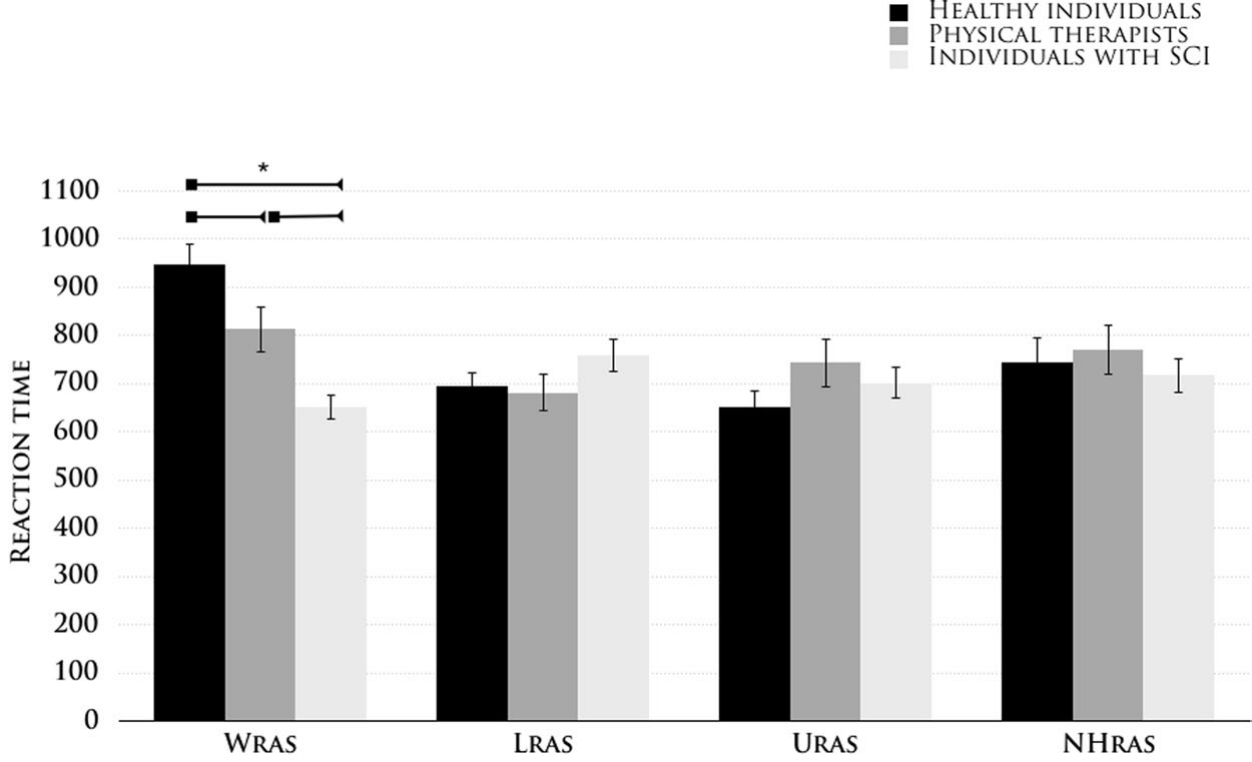
Latency in action-sound discrimination. The mean latency for each sound category (upper (U_RAS_) and lower (L_RAS_) limb-related action sounds, wheelchair-related action sounds (W_RAS_), and animal action-related sounds (NH_RAS_)) in the three subject groups (healthy individuals, physical therapists and individuals with spinal cord injuries). The error bars indicate the standard error of the mean (SEM). The asterisk (*) indicates significant results from the post hoc comparisons (p < 0.05).

The ANOVA of latency (Fig. 2) revealed no significant main effects of group (*F*_2,57_ = 0.85, p = 0.43) but a significant effect of sound category (*F*_3,171_ = 8.3, p = 0.0001, ηp^2^ > 0.13) and group × sound category interaction (*F*_6,171_ = 9.95, p = 0.0001, ηp^2^ > 0.26). SCI patient performance was similar for sounds related to actions of the lower (L_RAS_ = 760 msec) and upper (U_RAS_ = 703 msec) limbs (p > 0.19), suggesting that patients with SCI retained their upper and lower limb sound performance.

No significant latency differences were observed between the lower and upper limb sounds in any of the three groups: healthy subjects (L_RAS_ = 653 msec, U_RAS_ = 695 msec), physical therapists (L_RAS_ = 682 msec, U_RAS_ = 744 msec), and patients with SCI (all ps > 0.1). Importantly, the *post hoc* comparisons revealed that the patients with SCI discriminated the W_RAS_ earlier (652 msec) than the able-bodied individuals with comparable auditory experience (physical therapists: 813 msec, p < 0.01) and those with no comparable perceptual experience (healthy subjects: 949 msec, p < 0.0001). The latency difference in RTs between the physical therapists and healthy subjects was also statistically significant (p < 0.01). Notably, in patients with SCI, the RTs for W_RAS_ were comparable to the RTs elicited by upper (U_RAS_ = 703 msec, p > 0.26) but not of lower limb action sounds (L_RAS_ = 760 msec, p < 0.02). Regular wheelchair use contributed to a specific and significant readiness to recognize the sounds produced by a wheelchair. Latency improvements were not accompanied by changes in accuracy, thereby ruling out potential speed/accuracy trade-off effects.

There were also no differences between the three groups with regard to their responses to animal action sounds (p > 0.60). Moreover, no differences were observed in discrimination latency between the WHC_M_ and WHC_E_ sounds or with regard to the order of the presentation of the correct probe (all p > 0.80).

We also examined whether the time since the injury influenced RTs for sound discrimination. No significant correlations were found between the SCI lesion-testing interval and latency in the discrimination of each sound category (Spearman correlation analyses; L_RAS_, *r*_20_ = −0.06, *t*_18_ = −0.28, p > 0.78; U_RAS_, *r*_20_ = 0.04, *t*_18_ = 0.20, p > 0.84; W_RAS_, *r*_20_ = 0.12, *t*_18_ = 0.5, p > 0.62). All patients were in the chronic injury phase (at least six months post-injury), and the time since injury did not appear to play a major role in sound discrimination related to wheelchair action, suggesting that plastic changes could occur rapidly and lead to behavioral gain.

Altogether, these findings suggest that active use of a sound-producing device, as opposed to mere exposure to the sounds, modulates readiness to recognize associated sounds. The inability of patients with SCI to move their lower limbs did not influence their ability to discriminate sounds of lower limb movement.

### Subjective ratings of familiarity and perceived motor reactivity when listening to sounds

**Figure 3:**
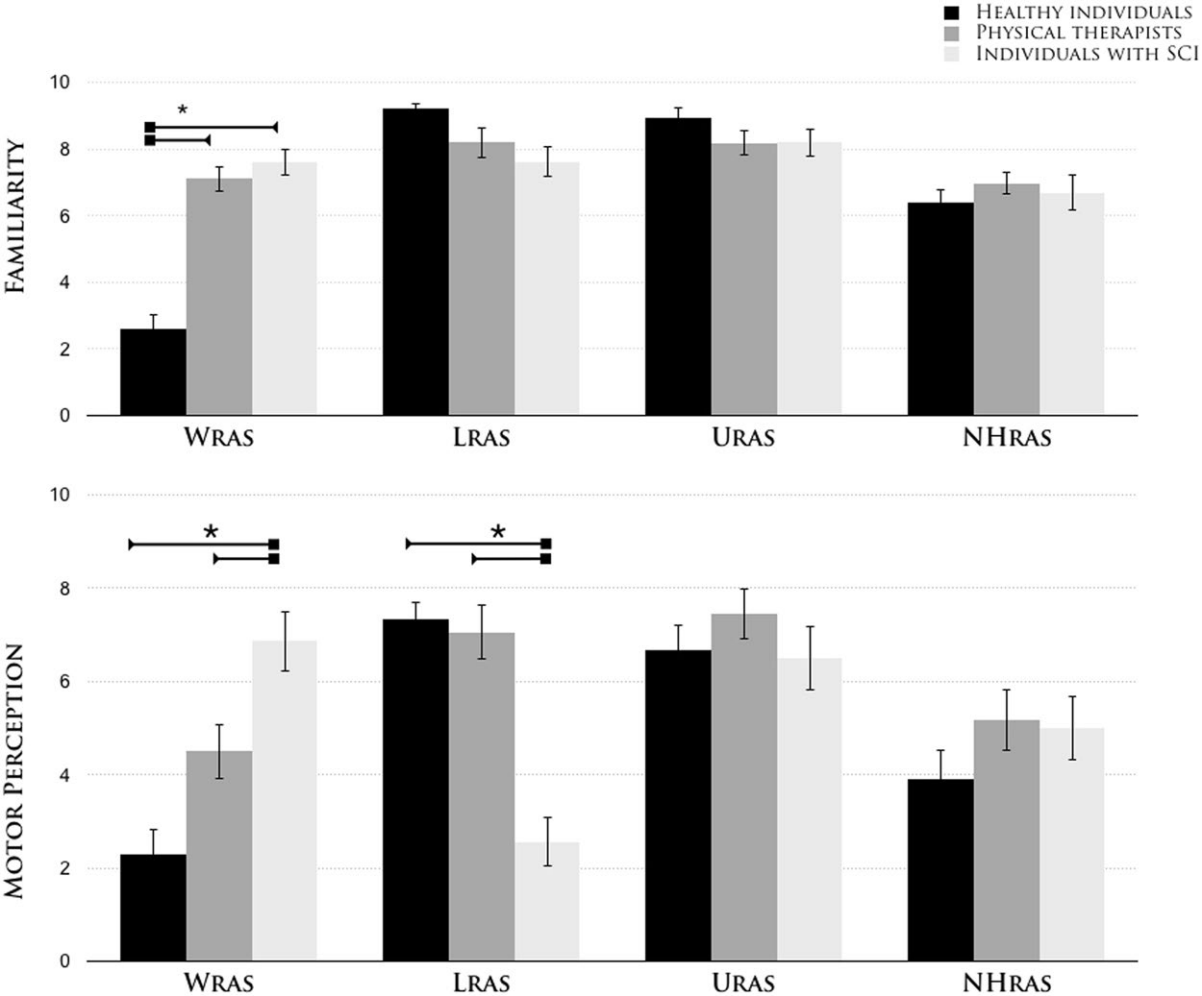
Subjective ratings of action-sound familiarity and perceived motor intensity. The mean subjective Visual Analog Scale (VAS) ratings for auditory familiarity and perceived movement for each sound category (upper (U_RAS_) and lower (L_RAS_) limb-related action sounds, wheelchair-related action sounds (W_RAS_), and animal action-related sounds (NH_RAS_)) in the three subject groups (healthy individuals, physical therapists and individuals with spinal cord injuries). The error bars indicate the standard error of the mean (SEM). The asterisks (*) indicate significant results from the post hoc comparisons (p < 0.05).

At the end of the test, a VAS was used to measure each participant’s perceived motor reactivity and auditory familiarity ratings for each of the four sound categories. The mean VAS ratings are shown in Fig. 3.

The ANOVA of perceived reactivity motor ratings for each sound (Fig. 3) yielded significant effects of sound category (*F*_3,171_ = 13.6, p < 0.0001, ηp^2^ > 0.19). *Post hoc* testing revealed that the subjectively perceived motor reactivity during passive listening was higher for human action sounds (L_RAS_ = 6.8, U_RAS_ = 5.6; p = 0.01) than for W_RAS_ (4.6; p < 0.02) or NH_RAS_ (4.7; p < 0.03). No significant differences were observed between groups (*F*_2,57_ = 1.61, p = 0.20). Crucially, the ANOVA revealed a significant group × sound category interaction (*F*_6,171_ = 16.02, p < 0.0001, ηp^2^ > 0.35). In patients with SCI, the perceived motor reactivity to sounds that implied lower limbs movement (2.6 ± 2.4; p = 0.0005) consistently received lower ratings than in able-bodied individuals (healthy individuals: L_RAS_ = 7.3 ± 1.7; physical therapists: L_RAS_ = 7.1 ± 2.7) and was significantly lower when compared with W_RAS_ sounds (6.9 ± 2.9) and U_RAS_ sounds (6.5 ± 3.04). These results suggest that the absence of motor signals reduces the reactivity with which actions can be perceived from an associated sound^48^. Instead, the perceived motor reactivity to WHC sounds received higher ratings in the patients with SCI (W_RAS_ = 6.9, p < 0.01) than in able-bodied individuals (healthy subjects: W_RAS_ = 2.3 ± 2.4; physical therapists: W_RAS_ = 4.5 ± 2.7).

Despite the aural expertise of physical therapists, their perceived motor reactivity to W_RAS_ sounds was significantly lower than their reactivity to human (U_RAS_ = 7.4 ± 2.5, L_RAS_ = 7 ± 2.7; p = 0.0001) and NH_RAS_ (5.1 ± 2.9; p = 0.002) action sounds. Unsurprisingly, healthy subjects were unaccustomed to the W_RAS_ sounds, and their perceived motor reactivity to them was significantly lower than their reactivity to the human (U_RAS_ = 6.6 ± 2.5, L_RAS_ = 7.3 ± 1.7; p = 0.0001) and was comparable to NH_RAS_ (3.9 ± 2.8; p = 0.16) action sounds. No significant differences in perceived motor reactivity were observed between the two groups of able-bodied individuals (p > 0.24).

The ANOVA of subjective familiarity ratings (Fig. 3) revealed a significant main effect of sound category (*F*_3,171_ = 34.8, p < 0.0001, ηp^2^ > 0.37). Specifically, higher VAS ratings were found for human action sounds (L_RAS_ = 8.4, U_RAS_ = 8.3) than for non-human action sounds (W_RAS_ = 5.7, NH_RAS_ = 6.7; p < 0.003). No significant differences in familiarity were observed between participant groups (*F*_2,57_ = 0.66, p = 0.42). However, we did observe a significant group × sound category interaction (*F*_6,171_ = 17.35, p < 0.0001, ηp^2^ > 0.37). Physical therapists (7.1 ± 1.7) and SCI patients (6.9 ± 2.9) had similar levels of familiarity with regard to W_RAS_ actions sounds (p > 0.37), and this level of familiarity was not significantly different from the familiarity with human lower limbs (physical therapists: L_RAS_ = 8.18 ± 2; SCI patients: L_RAS_ = 7.6 ± 2.3) and upper limbs (physical therapists: U_RAS_ = 8.17 ± 1.7; SCI patients: U_RAS_ = 8.2 ± 1.9) action sounds and non-human action sounds (physical therapists: NH_RAS_ = 6.9 ± 1.6; SCI patients: NH_RAS_ = 6.7 ± 2.4; all p > 0.14).

As expected, in the group of healthy subjects, the familiarity ratings for W_RAS_ action sounds (2.6 ± 1.9) were significantly lower than the familiarity ratings for the human (U_RAS_ = 8.9 ± 1.3, L_RAS_ = 9.2 ± 0.8; p = 0.0001) and non-human (NH_RAS_ = 6.4 ± 1.7; p = 0.0001) action sounds. The familiarity rating for W_RAS_ was significantly different from those measured in the physical therapists and SCI patients (all p < 0.0001).

The subjects were also briefly interviewed with regard to their feelings about the auditory stimuli. A “yes” or “no” response was required for the following questions: (1) “Do you pay more attention to a specific category of auditory stimuli?” and (2) “Do you feel more emotional participation when hearing a precise sound category?” In the case of a “yes” response, the subject was asked to explain the answer. Sound discrimination does not appear to be explained by category-specific, attention-driving tendencies. Indeed, all subjects stated that they did not pay particular attention to the specific sound category. Presenting the auditory stimuli in a random order may have prevented the subjects from focusing their attention on a particular auditory stimulus category. Four SCI patients declared an increase in emotional participation when hearing lower limb sounds, while another three reported greater emotional participation when hearing animal sounds. Only one SCI patient and one physical therapist (who was married to an individual with SCI) experienced more emotional involvement when hearing the WHC sounds compared with the other sound categories.

Together, these findings suggest that although the SCI patients and auditory experts (i.e. physical therapists) demonstrated the same degree of familiarity with the WHC sounds, the greatest differences between the three groups occurred with regard to the subjective motor experiences associated with WHC and lower limb action sounds.

## Discussion

Many theories have proposed an association between the perception and execution of actions, suggesting that both are coded according to a common representational format^49–55^. Neural studies in healthy^16,56,57^ and brain-damaged^58,59^ individuals indicate that action perception and execution rely on largely overlapping neural substrates. A lesser corticospinal motor reactivity to vision^24,25^ and sound of action^25,26^ in individual with congenital blindness and deafness, respectively, was found. Importantly, it is unclear whether lifelong (mobility by lower limbs) and newly acquired (mobility by WHC) perceptual and motor experiences differently impact the integrity of action-perception mapping. The present study investigated action sound mapping in SCI patients and revealed two key findings. First, SCIs that have induced a total loss of lower limb function do not lead to a general reduction of the perceptual motor mapping of lower limb action sounds. Second, we provided empirical measures revealing that the sounds related to their wheelchairs were more salient in patients with SCI than medical professionals who worked with wheelchair users.

### Multimodal coding of long-term action in the complete absence of motor mediation

Alteration of the action network involved in the perception of human motor actions may occur in the absence of a cortical lesion, as in blind^24,26^, deaf^25^ and SCI^28,60^ individuals. This prompted us to investigate whether somatosensory deafferentation and motor deefferentation of specific body parts alter the audio-motor mapping of actions generated by the affected body part. Thoracic and lumbar SCIs lead to a loss of movement in the legs while sparing arm function. Consequently, these types of injuries offer an ideal experimental approach for exploring how sound-related actions associated with the upper and lower limbs are processed in an individual.

As mentioned previously, patients with SCIs exhibit reduced visual perceptual sensitivity regarding the biological motion of point-light displays of the entire body^28^ and specific impairments in the visual perception of form and action in disconnected body parts^27^. In this study, we expected that the processing of sounds depicting upper-limb actions would be unimpaired while the processing of sounds depicting lower-limb actions might be degraded. However, we obtained psychophysical evidence that paraplegic patients recognize lower- and upper-limb actions as efficiently as able-bodied individuals. Importantly, in SCI individuals, the perceptual ability to discriminate upper- and lower-limb action sounds was comparable. The inability to move and feel the lower limbs did not lead to a deficit in the sound discrimination of the actions, even several years after the initial injury.

Several mechanisms may explain the preservation of perceptual signaling referring to the paralyzed portion of the body following SCI. Even with a long history of absent sensation and movement after injury, accurate perceptual discrimination may be mediated by long-term motor representations that were learned before the injury. Studies of amputee patients have revealed that perceptual sensitivity associated with the missing limb remains accurate^61^, including in processes that require motor simulation^62^. Additionally, in clinical conditions such as the Möbius sequence, studies of expression recognition difficulties have provided evidence that feedback from facial movement is unnecessary, which is contrary to the strongest form of the embodied simulation theory^63^.

It is important to note that the sounds utilized in the present study are highly relevant to everyday motor functions that the patients had performed regularly prior to injury. These representations could, however, be updated and reinforced through visual and acoustic experiences involving ambulatory individuals encountered in daily life. Action-related networks may be activated to mediate motor limb sound representation even if motor plans have not been utilized for years. Accordingly, the brain regions involved in foot movements appear to remain relatively preserved and active even years after the body has been massively deafferented/deefferented^64–69^.

All of the patients recruited into our study were also involved in a motor program at a rehabilitation center. As part of the motor imagery program, they attempted natural movements and exercise training, including attempts at moving the foot and, to a lesser degree, walking. Accordingly, recent neuroimaging studies have demonstrated that a common observation-execution network including the ventral premotor cortex, parietal cortex and cerebellum is activated at a normal level through attempts to move a given body part and through observations of the movements of other individuals, long after the onset of complete SCI^67,68,70–72^.

Studies of either virtual (via transcranial magnetic stimulation) or natural lesions have probed the essential role of the fronto-parietal regions in mediating the auditory and visual processing of body actions^13,15,73–75^. The preservation of perceptual ability in SCI patients suggests that the cortical regions involved in action simulation could play a compensatory role by facilitating the maintenance of intact audio-motor resonance in patients with impaired lower-limb motor functions. However, the presence of intact audio-motor mapping in patients with profoundly impaired body-brain communication may conflict with findings from studies of visual-motor action translation in SCI patients^28,27^. One way to reconcile this potential discrepancy concerns the quality of the experience of actions mediated through visual vs. auditory inputs. Indeed, whereas vision allows one to directly simulate a specific action (e.g., grasping an object), auditory input may elicit the simulation of more than one action related to the sound that was heard (e.g., clapping different hands), thus enabling the simulation of the heard action in multiple, indirect ways as well as enabling higher degrees of compensatory flexibility^19^. Importantly, although we used ecologically relevant sounds of daily human actions, perceptual alterations in SCI studies appear only when a more demanding task that requires the recognition of an unnatural expression (e.g., the direction of motion of a point-light^28^ or a humanoid form that assumes a sports posture^27^) is presented. Embodied simulation for most basic of perceptions could be not necessary^63^.

### Does the auditory coding of actions evoked by assistive tools trigger embodied simulation?

The present study investigated action sound mapping in individuals with SCI with functioning upper limbs and nonfunctioning lower limbs who have regained mobility using an assistive tool such as a wheelchair. We provide the first psychophysical evidence that patients with SCI can distinguish WHC sounds from other distracting sounds more rapidly than individuals with no direct perceptual or motor wheelchair experience. Notably, the ability of the audio–motor system to distinguish wheelchair actions recalls the greater perceived motor reactivity present when passively listening to WHC sounds. These findings suggest that the active use of a wheelchair—as opposed to a mere perceptual, passive exposure to it—modulates the readiness to recognize its associated sounds. Visual and auditory wheelchair familiarity, although not fundamental, certainly play a role in distinguishing its sounds, as indicated by the enhanced WHC sound discrimination of physical therapists when compared with healthy subjects. However, the acquisition of motor skills through physical vs. perceptual practice may imply a highly selective coupling of perceptual motor information.

Processing the sounds of a wheelchair may be involved in associating or matching motor ideas regarding upper limb manipulations that could be linked with sound production in order to set an accurate movement on wheelchair; this emphasizes the intimate relationships among perceptual motor systems. Thus, these patients’ ability to quickly extract and distinguish relevant WHC sounds may not be determined so much by familiarity with the signal characteristics of their assistive tool, but rather by the relationship between the sound and their experiences with the probable actions of wheelchair-executed motion^76^. Additionally, when a tool extends the movement potential of a physically impaired individual, it may be included in the internal representation of the body schema to meet the novel demands of immobile limbs, a phenomenon called tool embodiment. The striking effects of perceptual motor practice with specific objects have been shown to induce long-term structural changes in monkey^77^ and human^40,78^ body representations. Thus, the more rapid response to wheelchair sounds could be attributed to the integration of the signals (and their interactions) belonging to the person in terms of the active control and perception of the tool and the experience of the embodiment of the instrument into the motor abilities, including the perceptual features of the wheelchair^42,79,80^. As posited by previous theoretical^34,81^ and quantitative^38,41,82,83^ studies, the acquisition of wheelchair skills by SCI patients alters their body schema by adding corporeal awareness of the device. That is, tools that have been in contact with the body recalibrate multisensory representation^84,85^ and induce short- and long-term neuroplastic changes in the motor system^86^ following active (rather than passive) use of the body^87–89^. This means that the tool becomes part of the body in action and in the person in the sense that it modifies the way the person perceives^38^, moves in^90^, and relates to the world^34^. Although the process that regulates tool incorporation has different complexity levels, the embodiment and agency of devices could induce intuitive control and could facilitate learning, user efficiency, and the acceptance of new assistive device^7,12–14^. However, at present, embodied technology remains a concept that needs to be experimentally explored in order to forge new rehabilitative opportunities^33,40^. Accordingly, multimodal modulation—not only visual modulation—may be a viable intervention for tool rehabilitation and treatment following SCI^91,92^.

## Electronic supplementary material

List of the sounds of the auditory discrimination action task.

## Data Availability

Data that support the findings of this study are available from the corresponding author upon reasonable request.
