# Supplementary material for: Embodying functionally relevant action sounds in patients with spinal cord injury
Source: Sci Rep. 2018 Oct 23;8:15641. doi: 10.1038/s41598-018-34133-z (PMC6199269; doi:10.1038/s41598-018-34133-z)
Supplement: Supplementary file 1 — List of the sounds of the auditory discrimination action task. [file 41598_2018_34133_MOESM1_ESM.pdf]

Embodying functionally relevant action sounds in patients with spinal cord injury

Mariella Pazzaglia, Giulia Galli, James W. Lewis, Giorgio Scivoletto, Anna Maria Giannini and Marco Molinari

LIST OF SOUNDS

Upper Limb	Lower Limb	Wheelchair	Animals
Clapping hands, single human	Footsteps, man	WHCm being rolled down a hallway	Pigeon flying
Clapping hands, group	Footsteps, woman	WHCm being rolled, solid ground	Insects flying
Knocking on door	Running, hard surface	Keys opening door	Dog walking
Writing on chalkboard, quick	Running, solid ground	WHCm braking 1	Horses galloping, slow
Writing on chalkboard, slow	Running, group	WHCm braking 2	Horse galloping, solid ground
Scratching, quick	Footsteps, solid ground	Bicycle	Herd passing
Typing on computer keyboard	Going down wooden stairs, quick	WHCm rattle, cement surface	Horses trotting, soil
Typing on computer keyboard, fast	Going down stairs, hard surface	WHCm rattle, wooden surface	Horse trotting, quick
Drumming fingers	Footsteps, quick	Film projector	Cows moving
Scratching	Dancing, tapping fast	WHCm movement, slow	Birds flying
Scratching, fast	Dancing, tapping slow	WHCm rattle, wooden surface	Bird flying
Writing with chalk	Going up the stairs, quick	Zip fastener	Rattle snake rattling
Snapping fingers	Marching, group	WHCm rolling, indoor	Dog digging 1
Snapping finger	Marching, single human	WHCm rolling, hard uneven surface	Dog digging 2
Cracking fingers	Dancing, flamenco	Bowling ball	Dog footstep
Drumming fingers	Running, outdoor	WHCe braking 1	Horse galloping, slow
Drumming finger	Running, indoor	WHCe braking 2	Horse galloping, indoor
Typing on computer keyboard	Dancing, tip tap	Elevator	Dog running around
Cracking finger	Going up stairs, indoor	WHCe moving changing from high to lower speed.	Horses galloping, solid surface
Cracking knuckles	Going up stairs, outdoor	WHCe moving changing from a low to a higher speed	Horse gallop, solid surface
Snapping finger	Marching, slow	Coffee machine	Hoofed animal stampede
Hands drumming, quick	Footsteps, broken glasses	WHCe moving, solid ground	Bird flapping
Hands drumming	Footsteps, wooden surface	WHCe going back and forth	Butterfly flapping wings
Hands clapping	Going up stairs, man	Scooter	Horse trotting slow
Applause, medium crowd	Running, wet surface	WHCe turning on and accelerating fast	Horse galloping, indoor
Hands clapping, single human	Running, gravel	WHCe turning on and moving	Horses galloping, soil
Tapping fingers, multiple	Footsteps, wet surface	Hairdryer	Zebra trotting
Knocking on wooden door	Going up stairs, quick wearing tennis shoes	WHCe moving, solid ground	Bat flapping wings
Knocking on metal door	Going up wooden stairs, quick	WHCe accelerating	Hen flapping
Tapping fingers	Running, wearing tennis shoes	Scooter	Dog panting

Supplementary Material. List of the sounds of the auditory discrimination action task.
A list of the category of 10 sets of three sounds used in the matching-to-sample auditory discrimination action task. Each sound depicts upper (URAS) and lower (LRAS) limb-related action sounds, wheelchair-related action sounds (WRAS), and animal action-related sounds (AARS). In each category, the sample, the matching and the non-matching sound stimuli are indicated in the first, second and third rows of each of the 10 sound sets, respectively. The sounds were chosen on the basis of two psychophysics studies. During the test-creation phase, healthy subjects who did not subsequently participate in the study listened to each sound and assessed how easily it could be identified. The selection of the auditory stimuli was based on the results of a preliminary psychophysics study involving 20 healthy participants (11 men, 22–34 years of age). Each participant listened to each sound and chose between two verbal tags. Only one tag correctly described the sound; the other tag was used as a realistic distractor. Only the sounds that were correctly matched by at least 80% of the participants were used in the subsequent study. Ten groups of three sounds formed every trial set for each sound action category that was chosen for use in the final test. The test performances of 10 healthy participants (3 men; mean age, 23.6 ± 4.8 years) were assessed. No differences were observed in the recognition rate of the URAS (mean, 88%; range, 70–100%), LRAS (mean, 85%; range, 75–91%), WRAS (mean, 88%; range, 70–100%), or NHRAS (mean, 90%; range, 75–100%) action sounds ($P > 0.8$).
